# Ligand-Tuning of the Stability of Pd(II) Conjugates with Cyanocobalamin

**DOI:** 10.3390/ijms22157973

**Published:** 2021-07-26

**Authors:** Dominika Porębska, Łukasz Orzeł, Dorota Rutkowska-Zbik, Grażyna Stochel, Rudi van Eldik

**Affiliations:** 1 Faculty of Chemistry, Jagiellonian University, Gronostajowa 2, 30-387 Krakow, Poland; dominika.porebska@doctoral.uj.edu.pl (D.P.); stochel@chemia.uj.edu.pl (G.S.); 2 Jerzy Haber Institute of Catalysis and Surface Chemistry, Polish Academy of Sciences, Niezapominajek 8, 30-239 Krakow, Poland; dorota.rutkowska-zbik@ikifp.edu.pl; 3 Department of Chemistry and Pharmacy, University Erlangen-Nuremberg, Egerlandstr 1, 91058 Erlangen, Germany

**Keywords:** palladium(II) complexes, cyanocobalamin, drug delivery systems, complex stability

## Abstract

Besides the well-known functions performed by vitamin B_12_ (CblCN) in biochemical processes of the human body, an increasing interest has been raised by the possibility of its use as a transmembrane drug carrier, capable, among others, of enhancing the accumulation of inorganic cytostatics in cancer cells. The present study was aimed at determining the possibility of the formation of CblCN conjugates with Pd(II) complexes. A key aspect was their stability, which we attempted to tune by appropriate choice of ligands. Syntheses, spectroscopic analysis of postreaction systems and kinetic investigations of conjugate formation reactions, have been complemented by DFT modelling. The obtained results showed that ligand charge, geometry and electron affinity may have a significant impact on carrier binding and release leading to the activation of the Pd(II) complex. This provides a rationale to expect that with appropriate composition of the coordination sphere, it will be possible to extend the spectrum of less toxic inorganic chemotherapeutics.

## 1. Introduction

Participation of vitamin B_12_ in important life processes, came to human consciousness over a century ago, before the compound was structurally and functionally defined [1]. Nowadays, we know that the particular structure of this compound, the properties of both the central ion and the macrocyclic ligand, make cobalamin (Cbl) unique and predispose it to accomplish special tasks primarily as a precursor of methylation coenzymes, which is an important step in the regulatory processes of erythrocyte production, amino acid metabolism, DNA synthesis, etc. [2,3]. Detailed recognition of the nature of its interactions with axial ligands as well as biopolymers, particularly transport proteins, has raised a rapid growth of interest in B_12_-inspired compounds in the context of diverse biomedical applications [4]. Hence, it is used as cyanide detecting [5] and detoxifying agent [6], and an increasing number of derivatives are applied as anti-vitamins in the regulation of pathological biochemical processes [7] or as activators of enzymes, e.g., guanylyl cyclase [8]. During the last several years, the concept of using cyanocobalamin as a carrier of agents used in targeted diagnostics and therapy, has also been strongly developed [9].

Trends in the development of cobalamin derivatives for medical applications were determined by the intended intracorporeal mechanisms of action. Their common denominator, however, almost always involves the fact that rapidly proliferating cells have an enhanced demand for vitamin B_12_ [10,11]. Its cellular uptake is a highly sophisticated process occurring through interactions with three different transporting proteins, namely intrinsic factor, haptocorin and transcobalamin II [2]. The structures of complexes that Cbl forms with proteins on passage through the cell membrane reveal their complexity, which is due to the involvement of many weak interactions [12,13]. However, it was demonstrated that certain changes in these interactions do not have a significant effect on the recognition of the vitamin by the receptor [14]. The β-axial ligand and the ribose residue at the dimethylbenzimidazole group coordinating the Co(III) ion at the α-position, proved to be particularly useful sites in the context of attachment of a therapeutic or diagnostic agent [15]. Taking advantage of this fact, a wide range of vitamin B_12_ derivatives with different metal complexes have been proposed. The ability to be attached to a transmembrane carrier is particularly important for metal compounds that cannot penetrate it on their own, such as technetium and rhenium, whose radioisotopes (^95m^Tc as well as ^186^Re and ^188^Re) are commonly used in radiotherapy and radiodiagnostics [16]. Their stable binding to the carrier is ensured by specialized chelate groups introduced by modification of the side substituent in the corrin ring [17,18]. Stable binding to the carrier is usually expected for diagnostic agents. The ability of Cbl to selectively accumulate in cancer tissue has been used successfully in NMR imaging using gadolinium complexes as contrast agent [19]. An even tighter covalent bond binds the carrier to a number of fluorescent markers [20,21]. Such conjugates provide significant benefits, for example, in the surgical resection of malignant tumors [22].

In the context of therapeutic applications, the predominant structure is one in which the biologically active agent is attached to the cobalamin Co(III) ion via a bridging ligand [23,24,25]. Such an arrangement of the molecule has the primary advantage of being able to release the drug once it crosses the cell membrane barrier. The cyanide group in the natural form of vitamin B_12_ is sufficiently predisposed to perform this function due to the presence of a free donor nitrogen atom. In addition, as shown by Alberto and coworkers, the CN-bridged complex is stable in serum, whereas in the cell it breaks down by the action of ATP-dependent adenosylation enzymes that reduce the Co(III) ion [23,26,27]. Alternatively, light can be used as a trigger to break the carrier–drug bond. This strategy has an additional advantage as it gives control over the release of the drug. An interesting example is simply hydroxocobalamin, whose illumination after accumulation in a tumor, leads to homolytic cleavage of the Co–OH bond [28]. Thus, the released hydroxyl radicals are capable of cleaving DNA strands. The light access-controlled stability of the adducts is further exploited in strategies for the delivery of therapeutic doses of carbon monoxide in so-called CO-releasing molecules (CORMs), in which the appropriate photons strip the carbonyl ligands off the Cbl-conjugated Re [29] or Mn [30] complexes.

By using CblCN as a carrier, inorganic cytostatic agents can also be selectively accumulated in cancer cells, so that the side effects of traditional chemotherapy are significantly reduced. For obvious reasons, most of the studies reported have focused on cisplatin and related complexes [23,25,26,27,31,32]. Efficient inhibition of DNA replication and transcription abilities, requires a drug release within the cell that is enabled by CN bridging, which is prone to the action of both intracellular enzymes [23] and visible light inducing photo-decay of the cobalt-carbon bond [33]. The slight reduction in cytostatic activity of Pt(II) complexes in CblCN conjugates is compensated for by the greatly improved cell targeting.

On the other hand, there are also some undesirable effects of excessive stability of Cbl-drug adducts. They reveal themselves by the accumulation of these toxic compounds in the liver and kidneys, which are the natural sites for vitamin B_12_ storage [10,11].

Aware of the vital importance of the appropriate stability, we synthesized model adducts of CblCN with several Pd(II) complexes. We aimed to find out how the diverse nature of ligands, both in terms of electron and steric effects, is reflected in CN bridging properties. Spectroscopic and kinetic studies are supported by modelling of adduct structures based on DFT calculations. With this approach, we were able to find hints on the selection of ligands in cytostatic Pd(II) and Pt(II) complexes for optimal therapeutic effect using CblCN conjugates.

## 2. Results and Discussion

### 2.1. Formation of CblCN-Pd(II) Conjugates

**Figure 1:**
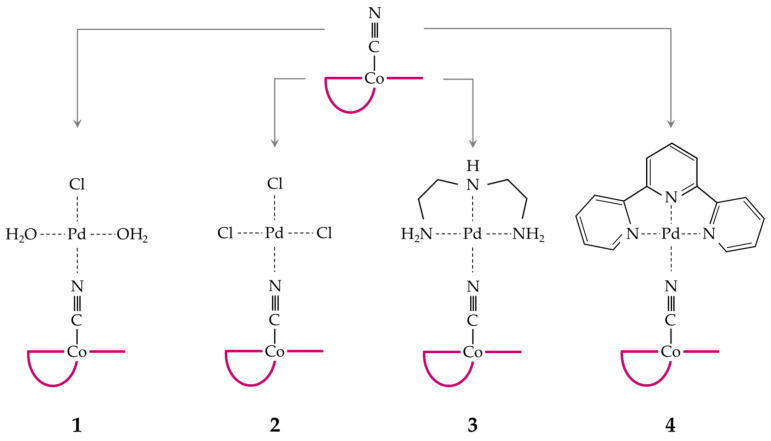
CblCN conjugates with Pd(II).

Four binuclear complexes (Figure 1) containing CblCN and various coordination forms of Pd(II), were synthesized. Two species with a mixed set of chloro and aqua ligands (structures **1** and **2** in Figure 1) were supplemented by two having tridentate ligands of diethylenetriamine (dien; **3**) and terpyridine (terpy; **4**). They were all conjugated to CblCN via the nitrogen atom of the cyano group substituting the solvent molecule in the conjugated complex.

**Figure 2:**
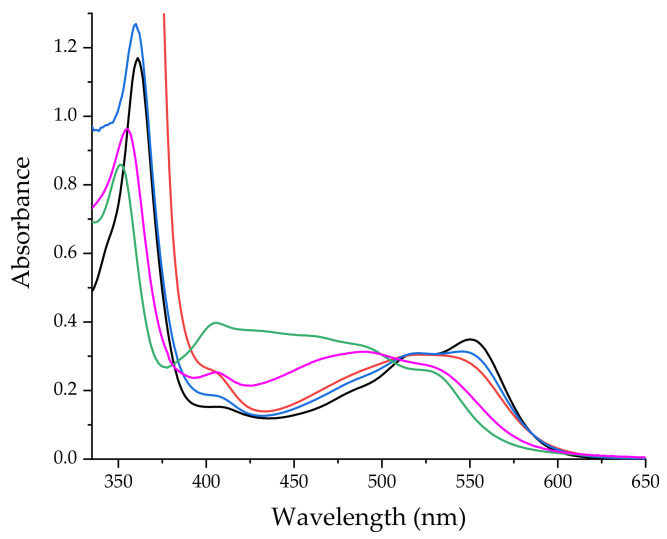
UV-Vis spectra of CblCN (black) and its conjugates with Pd(II) complexes: **1** (pink), **2** (green), **3** (blue) and **4** (red).

The formation of conjugates induces considerable changes in the electronic spectrum of CblCN, which applies to both the β- and γ-bands, undergoing a pronounced hypsochromic shift (Figure 2).

There are large spectral changes for compounds **1** and **2**, compared to much smaller spectral changes for compounds **3** and **4**. Thus, in the case of compounds **3** and **4** the binding constant is much lower than in the case of compounds **1** and **2** as clearly seen from the observed spectral changes. Therefore, in the case of compounds **3** and **4** a much larger excess of Pd(dien)^2+^ and Pd(terpy)^2+^, respectively, will be required to form the bridged complex.

A more detailed description of the formation of the individual conjugates, their kinetic analysis and spectroscopic characteristics of the products obtained, are presented below.

**Conjugate 1.** Ammonium tetrachloropalladate(II) was stabilized in water at pH = 3 and 2, which resulted in conditions where the predominant species are [PdCl_2_(H_2_O)_2_] and [PdCl(H_2_O)_3_]^+^, respectively. Reactions of both complexes with CblCN were performed and the preliminary spectroscopic observations showed that lower pH offers considerably faster conjugate formation. In addition, a strong dependence of the reaction yield on the excess Pd(II) used was found, indicating an equilibrium system with a significant contribution from the back reaction. Therefore, the synthesis of **1** and subsequently the other conjugates, was carried out at the ratios of [Pd(II)]:[CblCN] = 100. At pH = 2 the final spectrum was characterized by absorption bands at 406, 489 and 527 nm (pink line in Figure 2). Formation of **1** was confirmed by mass spectroscopy, which provided the spectrum including the peak around 374 *m/z* resulting from the isotopic distribution of Pd(II). This is considered as evidence for the formation of **1** (green line in Figure S1 in Supplementary Materials).

**Figure 3:**
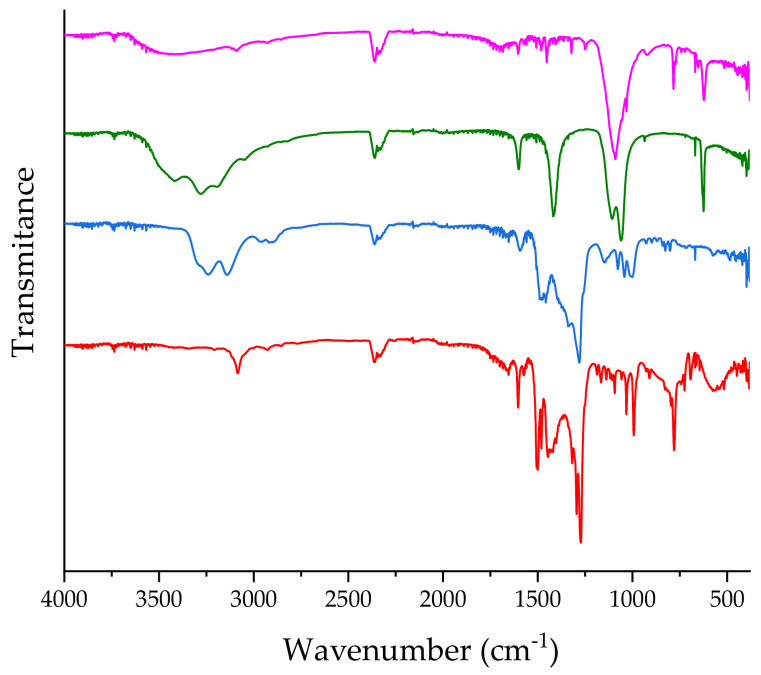
The ATR-IR spectra of the reaction mixtures containing CblCN-Pd conjugates: **1** (pink), **2** (green), **3** (blue) and **4** (red).

Formation of **1** was confirmed also by changes in the infrared spectrum (pink line in Figure 3), occurring in the range characteristic for the stretching Co–C vibrations [34], namely the shifts from 496 cm^−1^ to 515 cm^−1^.

Definitive confirmation of conjugate formation was provided by ^15^N-NMR spectra. In the latter case, CblCN with 100% labelled nitrogen atom of the cyano group, was used in the synthetic step of the ^15^N-labelled complex. The reference compound was ^15^NH_4_Cl (20 ppm). A band shift for complex **1** relative to the band for cobalamin is 86 ppm (Figure S2).

**Figure 4:**
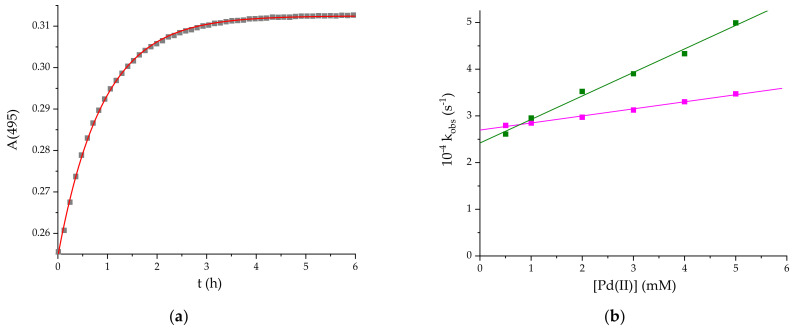
(**a**) The kinetic trace at 495 nm for the reaction of [PdCl(H_2_O)_3_^+^] and [CblCN] (10:1) and (**b**) the dependence of k_obs_ on Pd(II) concentration for the formation of **1** (pink) and **2** (green). pH = 2, 25 °C, [CblCN] = 10^−4^ M.

The kinetics of the formation of **1** was followed at pH = 2, 25 °C under pseudo-first-order conditions with 10- to 100-fold excess of [PdCl(H_2_O)_3_]^+^. The kinetic traces (see Figure 4a) were fitted with monoexponential functions and the derived k_obs_ values were plotted against Pd(II) concentration (pink series in Figure 4b). The linear relationship has a large intercept, thereby confirming the significant contribution of the back reaction in (1).

(1) $$[\mathrm{PdCl}(H_{2}O{)}_{3}{]}^{+}+\mathrm{CblCN}\;\begin{array}{c} k_{1}\\ ⇄\\ k_{-1} \end{array}\;[\mathrm{CblCN-PdCl}(H_{2}O{)}_{2}{]}^{+}+H_{2}O$$

According to the course of the concentration dependence (Figure 4b) the reaction follows the rate law (2):

k_obs_ = k_1_[Pd(II)] + k_−1_ (2)

for which k_1_ = (1.51 ± 0.06) × 10^−2^ M^−1^s^−1^ and k_−1_ = (2.69 ± 0.02) × 10^−4^ s^−1^ refer to the formation and decay of the conjugate, respectively. As a result the overall equilibrium constant K = k_1_/k_−1_ = 56 ± 3 M^−1^.

**Conjugate 2.** The reaction in MeOH was carried out using the same reactants as for the reaction in water, which led to the formation of **1**. However, based on literature data [35], it was found that the predominant species of Pd(II) is [PdCl_3_MeOH]^−^ as evidenced by the absorption bands at 324 and 427 nm. Following the above described procedure, the reaction with CblCN was performed in 100-fold excess of Pd(II). The reaction progress was accompanied by the decrease in absorbance at 520 and 550 nm as well as formation of new bands at 404 and 488 nm (green line in Figure 2). The mass spectrum of the post-reaction mixture showed bands around 391 *m/z* thus providing evidence for the formation of **2** (blue line in Figure S1). The infrared spectrum (green line in Figure 3) recorded in the postreaction mixture revealed a 7 cm^−1^ shift of the frequency of Co–C stretching vibrations.

The kinetic studies on the formation of **2** were performed at 25 °C under pseudo-first-order conditions at [Pd(II)]:[CblCN] ratios ranging between 10 and 100. The k_obs_ values obtained by fitting the kinetic traces at 470 nm with mono-exponential functions increased proportionally with Pd(II) concentration, as shown in Figure 4b (green series). The intercept in this relationship was again significant, although, because of the faster increase in k_obs_ with Pd(II) concentration, its contribution to the equilibrium state was somewhat smaller than in the formation of **1**.

Hence the reaction (3):

(3) $$[{\mathrm{PdCl}}_{3}(\mathrm{MeOH}){]}^{-}+\mathrm{CblCN}\;\begin{array}{c} k_{1}\\ ⇄\\ k_{-1} \end{array}\;[{\mathrm{CblCN-PdCl}}_{3}{]}^{-}+\mathrm{MeOH}$$

follows the rate law given in Equation (2), and the k_1_ and k_−1_ values are (5.04 ± 0.02) × 10^−2^ M^−1^ s^−1^ and (2.42 ± 0.07) × 10^−4^ s^−1^, respectively, resulting in the equilibrium constant K = k_1_/k_−1_ = 208 ± 7 M^−1^.

**Figure 5:**
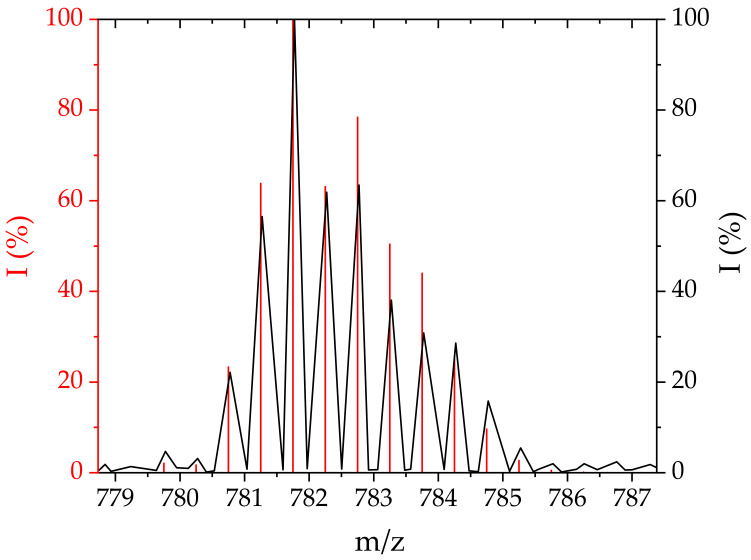
Mass spectra of **3** as recorded by ESI-MS in post-reaction mixture (180 °C, 0.4 Bar, 4.0 L/min) (black) and simulated using *The Isotope Distribution Calculator* (red).

**Conjugate 3.** CblCN-Pd(dien) was obtained from the reaction of CblCN with [Pd(dien)H_2_O]^2+^ carried out under ambient conditions in a 100-fold excess of Pd(II). The formation of the desired product was indicated by the decrease of the absorption bands at 550 nm and the formation of the new bands at 404, 520 and 545 nm (see the blue line in Figure 2). It was further confirmed by ESI-MS that **3** is indeed formed in solution, since the simulation carried out by considering the isotopic distribution of Pd(II) is in perfect agreement with the experimentally found spectrum (Figure 5).

Marked changes in the infrared spectrum in the fingerprint range of CblCN, namely the shift of the band from 496 cm^−1^ to 517 cm^−1^, indicate binding of CblCN (blue line in Figure 3).

The kinetics of the formation of **3** was followed spectrophotometrically at pH = 4.1 and 25 °C under pseudo-first-order conditions. The rate constants were determined for the reactions carried out in the range of 10- to 100-fold excess of [Pd(dien)H_2_O]^2+^. The kinetic traces (see Figure S3a) were fitted with monoexponential functions. A linear plot of k_obs_ versus [Pd(dien)H_2_O]^2+^ concentration has a significant intercept (see Figure S3b), which indicates the magnitude of k_−1_. Hence the reaction (4):

(4) $$[\mathrm{Pd}(\mathrm{dien})H_{2}O{]}^{2+}+\mathrm{CblCN}\;\begin{array}{c} k_{1}\\ ⇄\\ k_{-1} \end{array}\;[\mathrm{CblCN-Pd}(\mathrm{dien}){]}^{2+}+H_{2}O$$

is in agreement with the rate law given in Equation (2), in which at 25 °C, k_1_ = (11.9 ± 0.8) × 10^−2^ M^−1^ s^−1^ and k_−1_ = (11.1 ± 0.5) × 10^−4^ s^−1^. Hence the overall equilibrium constant K = k_1_/k_−1_ = 107 ± 12 M^−1^.

**Figure 6:**
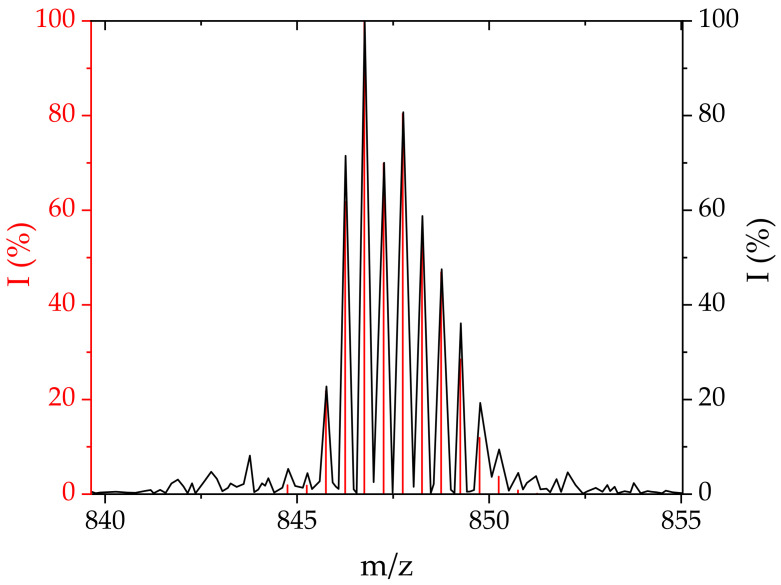
Mass spectra of **4** as recorded by ESI-MS in postreaction mixture (180 °C, 0.4 Bar, 4.0 L/min) (black) and simulated using *The Isotope Distribution Calculator* (red).

**Conjugate 4.** The terpyridine-containing **4** was obtained using [Pd(terpy)H_2_O]^2+^ as the substrate. This complex was prepared by induced aquation of the chloride complex, which was found to be nonreactive towards CblCN. The reaction with CblCN was carried out at pH = 4.1, 25 °C, and 100-fold excess of Pd(II). The UV-Vis spectrum of the postreaction solution shows characteristic bands of **4** at 402 and 520 nm (red line in Figure 2). The formation of **4** was confirmed by the peaks around 847 *m/z* in the ESI-MS spectrum, which refer to the [CblCN-Pd(terpy)]^2+^ species (Figure 6).

Moreover, a >20 cm^−1^ shift in the frequency of Co–C stretching vibrations (red line in Figure 3) indicates substitution of a water molecule in the Pd(II) complex with CblCN.

**Figure 7:**
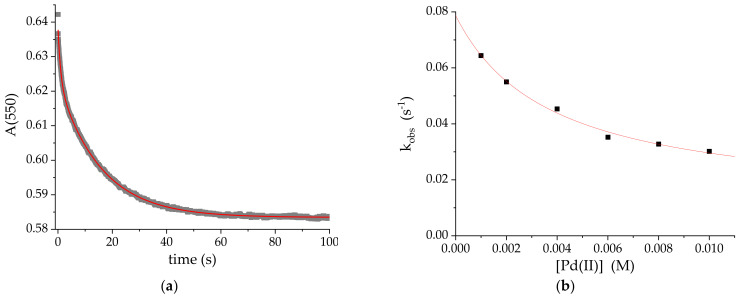
(**a**) Kinetic trace at 550 nm at 10:1 concentration ratio and (**b**) the concentration dependence for the formation of **4**. T = 25 °C, pH = 4.1.

The kinetics of the formation of **4** was studied using a single wavelength mode of stopped-flow technique. The experiments were carried out in 10- to 100-fold excess of [Pd(terpy)(H_2_O)]^2+^ at 25 °C and pH = 4.1. Kinetic traces recorded at 550 nm had essentially the character of single-exponential decays, however, for lower concentrations of the Pd(II) complex an additional component could be distinguished (Figure 7a), too fast for the stopped-flow technique. Since its contribution faded completely with increasing concentration, this component was omitted from the kinetic analysis.

The k_obs_ value for the actual step of conjugate formation decreases to reach the minimum value at infinite [Pd(terpy)H_2_O]^2+^ concentration (Figure 7b) and thus corresponding to the rate law (5):

(5) $$k_{\mathrm{obs}}=\frac{k_{1}k_{2}\left[\mathrm{Pd}\left(\mathrm{II}\right)\right]+{\;k}_{-1}k_{-2}}{k_{2}\left[\mathrm{Pd}\left(\mathrm{II}\right)\right]+{\;k}_{-1}}$$

which involves two subsequent equilibrium reactions with forward and backward reaction rate constants k_1_, k_−1_ and k_2_, k_−2_, respectively. We suggest that the relatively fast step is a water exchange reaction (6):

(6) $$[\mathrm{Pd}(\mathrm{terpy})H_{2}O]2^{+}+H_{2}O^{*}\;\begin{array}{c} k_{1}\\ ⇄\\ k_{-1} \end{array}\;[\mathrm{Pd}(\mathrm{terpy})H_{2}O^{*}]2^{+}+H_{2}O$$

that precedes the actual conjugate formation step (7):

(7) $$[\mathrm{Pd}(\mathrm{terpy})H_{2}O^{*}]2^{+}+\mathrm{CblCN}\;\begin{array}{c} k_{2}\\ ⇄\\ k_{-2} \end{array}\;[\mathrm{CblCN-Pd}(\mathrm{terpy})]2^{+}+H_{2}O^{*}$$

At pH = 4.1 and 25 °C, the “on” and “off” rate constants for reaction (6) are 1.11 × 10^−2^ s^−1^ and 1.56 × 10^−2^ s^−1^, while for reaction (7) they are 4.15 M^−1^ s^−1^ and 7.87 × 10^−2^ s^−1^, respectively.

### 2.2. Mechanistic Considerations

The formation of binuclear complexes such as CblCN-Pd(II) conjugates usually occurs by using a bridging ligand that is part of the coordination sphere of one of the metal centers. Therefore, from a mechanistic point of view, they should be regarded as ligand substitution reactions, which in the present situation become the complex providing the donor group. For square-planar complexes, typical of Pd(II) as well as Pt(II), this mechanism is almost always associative, due to the easy access to the central ion via the free dz_2_ orbital. The choice of intermediate geometry (tetragonal pyramidal or trigonal bipyramidal) as well as the ease with which it can be converted to the initial state by releasing the redundant ligand, strongly depends on the composition of the coordination sphere. Both the charge of the ligand and the number of coordination bonds formed, are of importance. In the context of medical applications, all of these factors are important in both the formation of conjugates with the carrier, their degradation in the cell and also DNA binding.

To test the effect of ligands on the stability of CblCN-Pd(II) conjugates, we selected four systems in which Pd(II) is coordinated by monodentate anionic and neutral ligands (**1** and **2**) and tridentate aliphatic (**3**) or aromatic (**4**) chelators.

The direct use of tetrachloropalladate(II) as a substrate in solution provides a whole palette of complexes with varying reactivity, depending on the properties of the medium. The final composition of the predominant species, results from the donor properties of the solvent, Solv:Cl^−^ concentration ratio, pH, etc. Preliminary tests enabled to determine the optimized conditions indicating pH = 2 as the most favorable for the formation of a conjugate with CblCN. At lower H^+^ concentrations, the reaction slowed down so much that only slight progress was observed at pH = 3 over a period of 12 h. This effect is clearly due to the change in speciation. At pH = 3, it is [PdCl_2_(H_2_O)_2_], whereas, at pH = 2, the predominant species is [PdCl(H_2_O)_3_]^+^ [35,36]. The reaction is hence greatly accelerated by electrostatic interactions enhanced by the effective charge of the complex. It is further favored by the higher number of labile ligands which release is rate-limiting for the second step of conjugate formation. Despite this, the very large intercept in the k_obs_ dependence on Pd(II) concentration (Figure 3b) indicates a significant contribution of the back reaction, which accounts for the relatively low value of the stability constant of **1** (K_1_ = 56 ± 3).

Despite the similarity in ligand nature of water and methanol, expressed in comparable values of both donor number [37] and pK_a_ [38], clear differences could be observed in the kinetics of formation of CblCN conjugates with chloride Pd(II) complexes in both solvents (k_1_^MeOH^ ≈ 5 × k_1_^water^). The main reason for this effect is the initial composition of the Pd(II) complex. Surprisingly, the complex formed in MeOH contains more chloride ions, so that the effective charge would seem to be unfavorable for the formation of the encounter complex. Since the k_2_ values in both solvents are almost identical, the Cbl-Pd(II) conjugate containing chloride ligands is almost four-fold more stable in MeOH.

The kinetics of ligand (L) substitution reactions in Pd(dien)L complexes have been extensively studied during earlier decades [39,40]. In general, these reactions are fast, and although they all follow an associative mechanism some differences are observed, which are related, among others, to the properties of the leaving ligand [41]. This accounts for the need to force the aquation reaction of [Pd(dien)Cl]^+^, which was found to be nonreactive towards CblCN. The water molecule is readily released from [Pd(dien)H_2_O]^2+^ as demonstrated by the relatively high k_1_ value. By facilitating the release of the auxiliary ligand diethylenetriamine, however, induces also a weakening of CblCN binding, so that the stability constant of the conjugate is between the K values for the chloride complexes in water and methanol.

The formation of the conjugate involving a terpyridine ligand appeared to be a particularly interesting case. Similarly to the dien system, the Pd(II) complex with terpy accompanied by the chloride ligand did not bind to CblCN. The reaction of [Pd(terpy)H_2_O]^2+^ with CblCN took less than 3 min, prompting the study of its kinetics using the stopped-flow technique. The decreasing concentration dependence, expressed by Equation (5), resembles, among others, the previously reported rate law for the reaction of N-methylimidazole with aquacobalamin, Cbl(H_2_O) [42]. However, since the proposed type of mechanism for the formation of the 2:1 complex cannot be realized in our system, we consider here rather competition of two ligand substitution reactions on Pd(II). The strong π-acceptor properties of terpy make Pd(II) an exceptionally strong electrophile, which clearly accelerates the coordination of all ligands available in this system. Consequently, the rate of decay of the conjugate **4** (k_−2_) exceeds the rate of its formation reaction, which is limited by the rate of the water exchange reaction (k_1_).

As an alternative to Equation (6), one may consider a reaction involving Cbl(H_2_O), some fraction of which may appear in the system due to the large excess of solvent over CblCN as shown in (8):

(8) $$\mathrm{Cbl}(H_{2}O{)}^{+}+{\mathrm{CN}}^{-}\;\begin{array}{c} k_{1}\\ ⇄\\ k_{-1} \end{array}\;\mathrm{CblCN}+H_{2}O$$

However, the high stability constant of CblCN and the lack of spectroscopic evidence for Cbl(H_2_O) suggest a lower probability for such an interpretation [43,44].

### 2.3. DFT Calculations

Density functional theory (DFT) was employed to determine the geometric structure of the studied conjugates and their stability. The core of the corrin ring stripped of the peripheral groups, was used as a geometric model of the macrocyclic ligand in CblCN in which the dimethylbenzimidazole was replaced by an imidazole molecule. Such a model of cobalamin was already used in earlier studies and proved to yield reliable results [45,46,47]. For the conjugate **1** two different isomers were considered: *cis*- and *trans*- in respect to the location of the chloride ligand in the Pd(II) coordination sphere. Our calculations indicated that the *cis*-isomer has a slightly lower total energy (by 1.4 kcal/mol) and thus it would be the prevalent form of the **1**.

**Figure 8:**
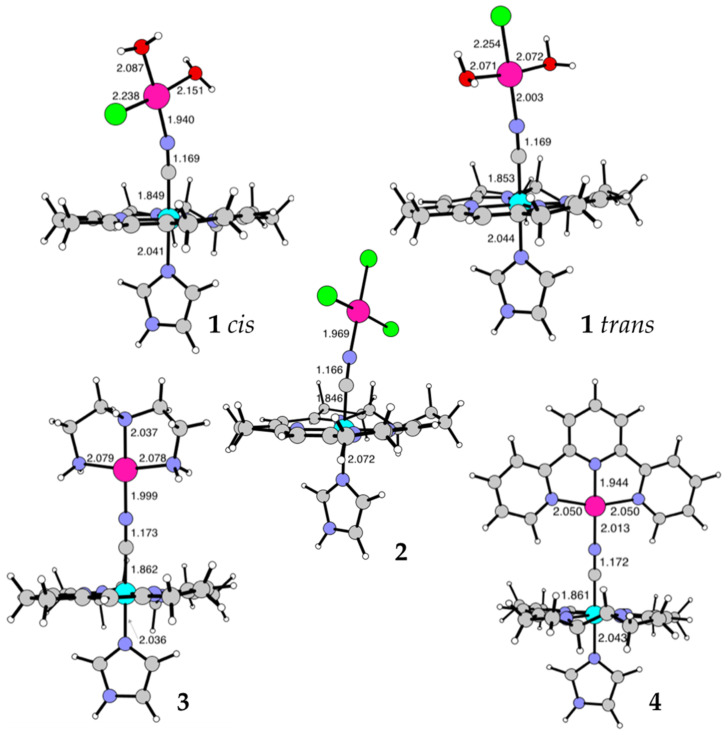
Geometric structures of the conjugates discussed in the current manuscript. Selected bond lengths are given in Å. Color code: C—grey, H—white, N—purple, Cl—green, Co—light blue, Pd—pink.

The geometries of the studied conjugates are depicted in Figure 8, while Table 1 gathers lengths of the bonds bridging the metal ions in the investigated structures, i.e., Pd–N, N–C, and C–Co. The computed C–Co and N–C bond lengths are comparable to those reported by Ruiz-Sánchez et al., for Cbl-CN-Pt(NH_3_)Cl_2_ (1.885 and 1.151 Å, respectively) [32].

The analysis of the geometric parameters of the studied conjugates allows for a clear differentiation between the structures in which Pd(II) is coordinated by small, monodentate ligands (Cl^−^ and H_2_O) and those in which it is chelated by larger species (terpy or dien). In the first group (**1** *cis* and **2**) the intermetallic distance is smaller than in the second group (**3** and **4**). The relatively long Pd-N distance in **1** *trans* is the demonstration of the trans-effect often observed in planar-square complexes. The radii of chlorine and water allows for relatively compact structures, in which the Pd–N, Co–C, and C–N bonds are short. The fact that the Pd–N, Co–C, and C–N bonds are considerably longer in dien- and terpy-containing conjugates may be related to steric hindrance caused by the macrocyclic system of CblCN and the large multidentate ligands coordinated to the Pd(II) ion. The longest Pd–N bond is found in **4** whereas the shortest in **1** *cis*.

The energy changes accompanying binding of the Pd(II) complexes to CblCN were computed in order to check the thermodynamic stability of the systems—see Table 2. In case of **1** the reaction starting from the PdCl_2_(H_2_O)_2_ complex is considered as indicated in (9):

(9) $${\mathrm{PdCl}}_{2}(H_{2}O{)}_{2}+\mathrm{CblCN}\;⇄\;\mathrm{CblCN-PdCl}(H_{2}O{)}_{2}+{\mathrm{Cl}}^{-}$$

In case of **2** the [PdCl_3_(MeOH)]^−^ complex as a substrate is proposed (see Equation (3)).

For conjugates **3** and **4** we assumed that they are formed in the reaction with the aqua complexes of palladium with CblCN as shown in (10):

PdL(H_2_O) + CblCN ⇄ CblCN-PdL + H_2_O (10)

The binding energies are calculated as the difference between the total energies of products and substrates. The negative binding energy values indicate that the formation of **2**, **3**, and **4** should thermodynamically be feasible. The formation of **1** is thermodynamically unlikely based on the data in Table 2. The values are relatively small and do not exclude the formation of **1** *cis* or **1** *trans*, but they indicate rather that the equilibrium between the PdCl_2_(H_2_O)_2_ and CblCN-PdCl(H_2_O)_2_ could be shifted towards substrates. This result also explains why a large excess of PdCl_2_(H_2_O)_2_ is required to synthesize CblCN-PdCl(H_2_O)_2_.

## 3. Materials and Methods

### 3.1. Chemicals

Cyanocobalamin (CblCN), hydroxocobalamin hydrochloride (CblOH∙HCl), palladium(II) chloride, 2,2′:6′,2′-terpyridine, TRIS base and potassium cyanide-^15^N (KC^15^N), were obtained from Merck (Sigma-Aldrich, MI, USA). Ammonium tetrachloropalladate(II) was purchased from Alfa Aesar. Methanol (MeOH) and acids (hydrochloric acid 35–38%, nitric acid 65% and perchloric acid 70%) were obtained from Chempur. All chemicals were of at least analytical grade.

### 3.2. General Procedure

The aqueous solutions were prepared in water deionized with Hydrolab HPL10 UV system. The pH of solutions was set with HClO_4_ or simply adjusted with the concentrated acid and controlled with micro pH combination electrode (Sigma Aldrich) filled with 3 M KCl/saturated AgCl solution combined with CP-401 pH-meter (Elmetron, Zabrze, Poland). The concentration of CblCN was determined from the molar absorption coefficient. All reactions were performed in the presence of air under ambient conditions.

### 3.3. Syntheses of the Palladium(II) Complexes

#### 3.3.1. [PdCl_n_(Solv)_4−n_]^2−n^

(NH_4_)_2_PdCl_4_ was dissolved in water to obtain a 1 mM solution, which was titrated with HClO_4_ to reach a pH = 2. Alteration of the Pd(II) speciation was monitored spectrophotometrically until the spectral changes were accomplished. Based on published spectra [36], [PdCl(H_2_O)_3_]^+^ was found to be the predominant stable species. Separately, (NH_4_)_2_PdCl_4_ was dissolved in MeOH to obtain a 1 mM solution and left at room temperature to establish the speciation equilibrium. Based on the UV-Vis spectra (absorption bands at 270 and 400 nm), [PdCl_3_(MeOH)]^−^ was recognized as the predominant species.

#### 3.3.2. [Pd(dien)(H_2_O)]^2+^

Synthesis of [Pd(dien)Cl]NO_3_ was carried out according to the published procedure [48,49]. In the first step 158 mg of PdCl_2_ was dissolved in 127 µL of DMSO. 2 mL of water was added and stirred in a closed glass flask, first for 1.5 h at 90 °C, and then at 40 °C. 0.10 M cold HCl was added to the mixture and stirred until the precipitation of [Pd(DMSO)_2_Cl_2_] was complete. The pale yellow product was filtered, washed successively with portions of cold water, ethanol and diethyl ether and air-dried. In the second step, 180 mg [Pd(DMSO)_2_Cl_2_] and 60 µL of diethylenetriamine in ca. 8 mL of methanol were mixed. The mixture was stirred in a glass flask under reflux overnight at 60 °C. About 30 mL of chloroform was added to separate the product out of solution. The resulting green-gray precipitate of [Pd(dien)Cl]Cl was filtered, washed with diethyl ether and air-dried. In the final step the 1 mM solution of [Pd(dien)Cl]Cl was titrated with AgNO_3_ at pH = 4.1, and the mixture was stirred for 4 h at 25 °C. The AgCl precipitate was filtered off from the [Pd(dien)H_2_O]^2+^ solution on a syringe filter.

#### 3.3.3. [Pd(terpy)(H_2_O)]^2+^

The corresponding terpy Pd(II) complex was obtained by a multistep reaction of potassium tetrachloropalladate(II) following the published procedure [50]. 18.3 mg of K_2_[PdCl_4_] was dissolved in 10 mL of water, filtered and mixed with 6 mL of 0.10 M HCl and 200 µL of 1,5-cyclooctadiene (COD). The mixture was stirred rapidly and heated to about 90 °C on a steam bath for 2 h and cooled down to 50 °C. After ca. 12 h the pale white crystals of [Pd(COD)Cl_2_] were filtered, washed sequentially with portions of water, ethanol and diethyl ether, and air-dried. In the second step, 150 mg [Pd(COD)C1_2_] was suspended in 10 mL of water and mixed with 94 mg of terpyridine. The mixture was stirred and heated at 50 °C for 5 h until a clear yellow-orange solution was obtained. During its subsequent cooling at room temperature, [Pd(terpy)Cl]Cl·2H_2_O precipitated, which was filtered off and air-dried. In order to remove chloride ions, [Pd(terpy)Cl]Cl·2H_2_O was dissolved in water and mixed 1:2 with AgNO_3_ at room temperature and filtered. The obtained solution of [Pd(terpy)H_2_O]^2+^ was used directly in reactions with CblCN.

#### 3.3.4. Adducts with CblCN

Synthesis of all adducts of CblCN with the different Pd(II) complexes were carried out at room temperature by mixing the solutions of the reactants in a volume ratio of 1:1. For maximum reaction yield, a 100-fold excess of Pd(II) was used. A differently optimized pH was used for each reaction system, viz. pH = 2 for CblCN-PdCl(H_2_O)_2_, MeOH for CblCN-PdCl_3_ and pH = 4.1 for CblCN-Pd(dien) and CblCN-Pd(terpy).

UV-Vis spectroscopy. The progress of the reactions was followed spectroscopically using Lambda 25 and Lambda 35 spectrophotometers (Perkin Elmer), both equipped with six-position cell holders and thermostated with Peltier PTP-6 modules. The UV-Vis spectra were recorded in 1 cm quartz cuvettes at 25 °C under ambient conditions.

Kinetic studies. The kinetics of slow reactions was studied using 0.88 cm tandem cuvettes and the spectrophotometers mentioned above. The kinetic traces were obtained either from repetitive scan spectra or single wavelength measurements. In the case of fast reaction, the kinetics was studied using SX20 stopped flow spectrometer (Applied Photophysics) equipped with both diode-array and a single wavelength detector, as well as Labo Plus (Polyscience) thermostatic bath. All experiments were performed at 25 °C under pseudo-first-order conditions. The data were analyzed using Pro-Data SX (Applied Photophysics) and Origin Lab software.

ESI-MS. The reaction products were identified from mass spectra recorded with tandem electrospray ionization mass spectrometer (ESI) and a quadrupole analyzer and a time-of-flight analyzer (microTOF-Q II, Bruker, Germany). The spectra were analyzed by comparison with simulations obtained using the Isotope Distribution Calculator and Mass Spec Plotter program.

ATR-IR. The vibrational spectra were recorded using FT-IR Spectrometer Frontier (Perkin Elmer) equipped with ATR lens ZnSe crystal. Spectra were collected from dried sample drops within the range of 380–4000 cm^−1^. The pressure force applied to the sample was optimized during the experiment.

NMR. ^15^N-NMR spectra were recorded using Bruker Avance III 600 MHz. ^15^N-labelled ammonium chloride was used as internal reference. In order to enable recording of an intense signal from the nitrogen atom of the CN group, we used CblCN obtained by mixing Cbl(H_2_O) and KC^15^N in a 1:1 molar ratio. All samples were prepared in 100% D_2_O following procedures given above.

DFT. Computational studies were performed using Density Functional Theory (DFT), as implemented in Turbomole V7.0.1 (TURBOMOLE, a development of the University of Karlsruhe and Forschungszentrum Karlsruhe GmbH, 1989-2007, TURBOMOLE GmbH, since 2007; available online: http://www.turbomole.com (accessed on 7 February 2011). Gradient-corrected Becke-Perdew (BP) functional was used with def2-TZVP basis set for all atoms. The computations consisted of geometry optimization of the structures, further confirmed by vibrational analysis. Solvent effect was taken into account via COSMO approach using water (for conjugates **1**, **3**, **4**) and methanol (for conjugate **2**) as solvents. The standard convergence criteria for geometry optimization were applied, i.e., 10^−6^ a.u. for the energy, 10^−3^ for the gradient, and 10^−6^ for the root mean square of the density matrix.

## 4. Conclusions

The concept of using CblCN as a carrier to increase the cellular selectivity of inorganic cytostatic drugs has so far been developed almost solely for Pt(II) complexes. Pd(II)-based systems, although less common, are also applicable as potential chemotherapeutics [51]. Therefore, we attempted to demonstrate the possibility of obtaining CblCN-Pd(II) conjugates with controlled stability, providing a basis for predicting their fate along the pathway from drug administration to its interaction with the intracellular target.

The possibility of therapeutic applications of CblCN conjugates with Pt(II) or Pd(II) complexes depends on meeting several requirements such as: (i) sufficient stability in blood plasma, (ii) high degree of cellular uptake, (iii) cytostatic activity comparable to the free complex. Assuming that the carrier effectively delivers the drug to the selected cell, it becomes important that it does not prevent the interaction of the therapeutic agent with the DNA. Therefore, if the bulky carrier molecule makes a steric hindrance to intercalation, it is necessary to release it after crossing the cell membrane. Since it was demonstrated that CblCN-Pt(II) conjugates are efficiently decomposed inside cells by enzymatic reactions which lead to the generation of biologically active CblAdo and CblMe [23], one should assume that the same mechanism will be valid for Pd(II) species. A significant challenge is the much greater lability of Pd(II) complexes compared to Pt(II). Consequently, the question arises not whether the Pd(II) conjugates will decompose in the cell, but whether they will survive until delivered there. Obviously, the stability can be tuned to some extent by the composition of the coordination sphere. Our selection of such systems was intended to demonstrate how significant this effect is.

An important observation seems to be that the stability of the conjugate can be increased by increasing the number of chloride ligands relative to the water molecules. This effect is certainly related to the ligand charge. For chloride ligands, it is beneficial primarily owing to the high concentration of Cl^−^ in plasma. Its decrease on passage into the cell may therefore serve as an additional factor in conjugate destabilization. The expected substitution of Cl^−^ by H_2_O will also increase the probability of forming a species capable of cross-linking with DNA. When considering isomers of **1**, the *cis* species is slightly more stable. The difference is not particularly large, but the trend is in line with expectations in terms of possible medical application.

The usability of chelated Pt(II) complexes in chemotherapy is basically limited to systems with bidentate ligands that secure *cis* isomerism. More expanded chelators can impede interaction with DNA mainly by preventing the cross-linking. However, also such complexes show certain therapeutic activity, since even a single binding to DNA can still suppress its replication quite effectively. Our tested complexes with dien and terpy appear to be directly unusable assuming only such a mechanism of conjugate decomposition in which the cyano group remains bound to Pd(II). As model systems, however, they served their purpose, providing interesting information related to geometric and electronic effects. The dien-involving conjugate **3** is positively distinguished by both the magnitude of the binding energy and the rate constant of conjugate formation. However, the elongation of the entire bridging bond sequence is favorable for the destabilization of this system, which is also reflected in the high rate constant of the backward reaction. Consequently, the equilibrium constant is located in between values determined for **1** and **2**. In turn, the terpy conjugate **4** is distinguished by the most bulky surroundings of the Pd(II) ion, which, however, does not significantly affect the binding to CblCN by possible steric hindrance. On the contrary, due to the appropriate geometry of the chelate system, the angles between terpy or dien and the CN group are larger than 90°, which may facilitate the binding of such a large component as CblCN. Nevertheless, the most remarkable effect introduced by the use of terpy is the strong π-acceptor binding. It strongly influences the rate of exchange of an additional ligand, which in this case is a water molecule. Consequently, the competitive reaction becomes the limiting step in conjugate formation. The overall equilibrium constant for reaction (6) is 0.70, compared to 53 M^−1^ for reaction (7) at 25 °C. The latter value is much smaller for compound **4** than found for the other compounds **1** < **3** < **2** in that sequence, which is presumably due to the very efficient “off” reaction that is ca. 70 times faster than for compounds **1**, **2** and **3** as a result of the extreme lability of the terpy complex.

Conjugate **2** seems to be the most promising candidate from the point of view of potential therapeutic applications. This is supported primarily by its relatively high stability, which seems to be sufficient to maintain the bridging bond during transport into the cell. Important is the presence of chloride ligands, which is ensured by the high concentration of Cl^−^ in the plasma. The significant decrease in stability of **2** when transferred to the cytoplasm, will facilitate not only the dissociation of the Pd(II) complex, but also the formation of species capable of binding DNA. Thus, in this strategy, the high lability of Pd(II) complexes could provide an advantage over Pt(II) compounds.

## Figures and Tables

**Table 1:** Selected bond lengths in the CblCN-Pd(II) systems.

Conjugate	Pd-N [Å]	N-C [Å]	C-Co [Å]
**1** *cis*	1.940	1.169	1.849
**1** *trans*	2.003	1.169	1.853
**2**	1.969	1.166	1.846
**3**	1.999	1.173	1.862
**4**	2.013	1.172	1.861

**Table 2:** Binding energies of CblCN-Pd(II) conjugates.

Conjugate	E_bin_ [kcal/mol]
**1** *cis*	3.4
**1** *trans*	5.3
**2**	−7.5
**3**	−11.7
**4**	−10.1

## Data Availability

Not applicable.

## Supplementary Materials

The following are available online at https://www.mdpi.com/article/10.3390/ijms22157973/s1.
