# Supplementary material for: Ligand-Tuning of the Stability of Pd(II) Conjugates with Cyanocobalamin
Source: Int J Mol Sci. 2021 Jul 26;22(15):7973. doi: 10.3390/ijms22157973 (PMC8347314; doi:10.3390/ijms22157973)
Supplement: Supplementary file 1 [file ijms-22-07973-s001.zip › ijms-1309715-supplementary.pdf]

Supplementary Materials

Ligand-tuning of the stability of Pd(II) conjugates with cyanocobalamin

Dominika Porębska, Łukasz Orzeł, Dorota Rutkowska-Zbik, Grażyna Stochel and Rudi van Eldik

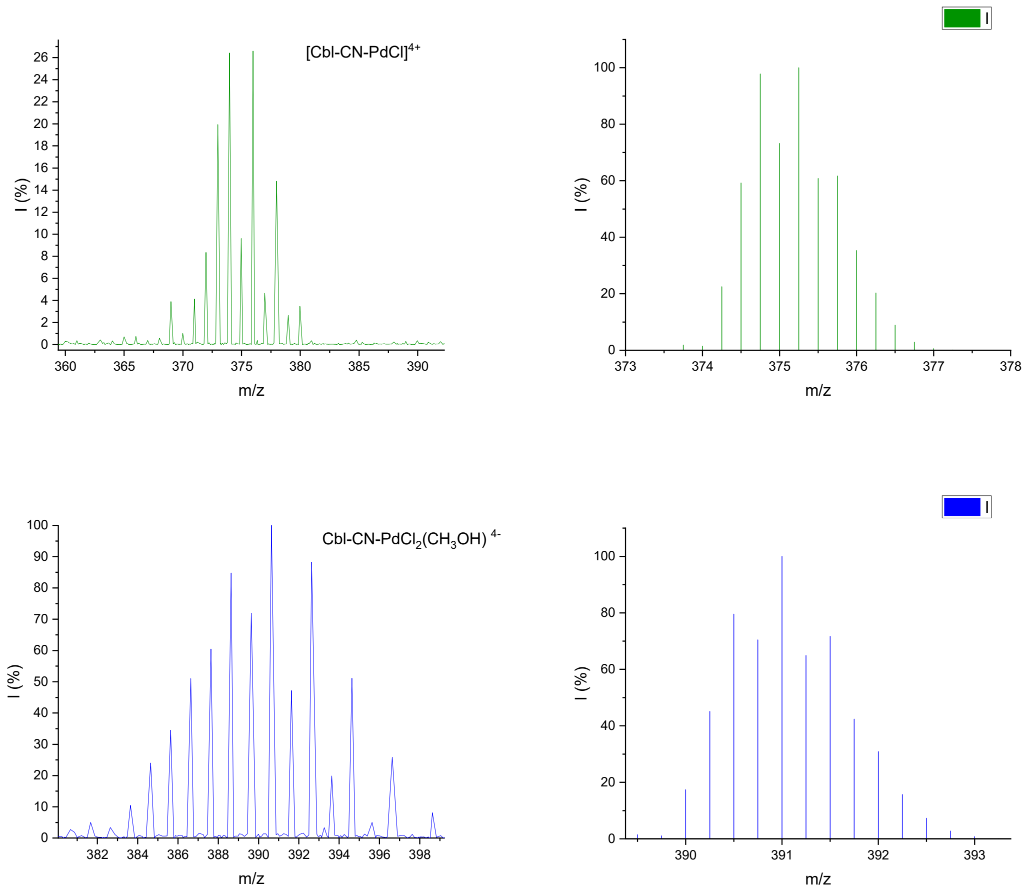

Figure S1. Mass spectra of **1** (green)**, 2** (blue) as (a) recorded by ESI-MS in post-reaction mixture (180 ^{o}C, 0.4 Bar, 4.0 l/min) and (b) simulated using *The Isotope Distribution Calculator*.

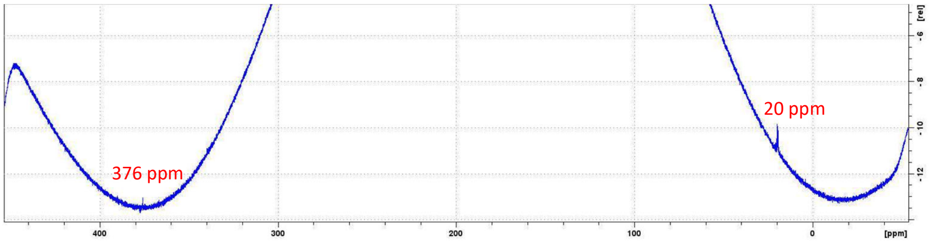

(a)

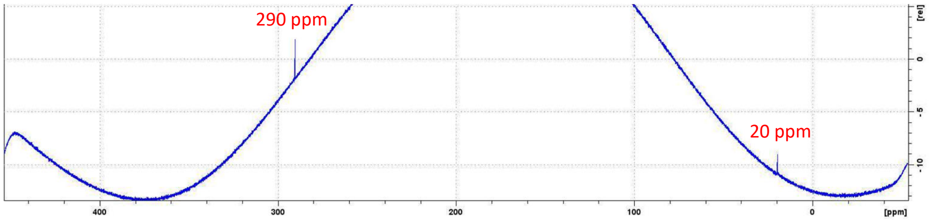

(b)

Figure S2. (a) The ^{15}N-NMR spectrum of the reaction mixture containing **1**, (b) the ^{15}N-NMR spectrum of CblCN.

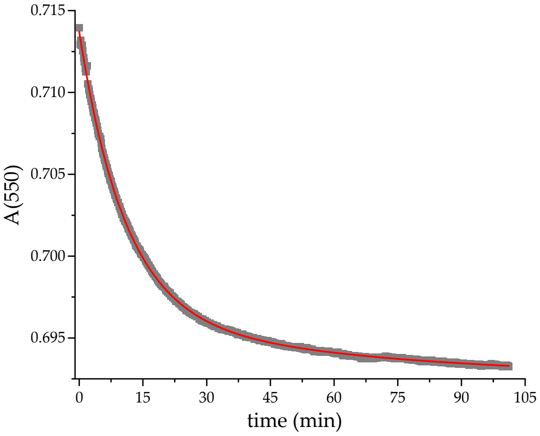

(a)

Figure S3. (a) The kinetic trace at 550 nm for the reaction of CblCN with $[Pd(dien)H_2O]^{2+}$ at 1:10 concentration ratio and (b) the plot of k_{obs} versus $[Pd(dien)H_2O]^{2+}$ concentration. [CblCN] = 1 x 10^{-4} M, pH = 4.1, T = 25 ^{o}C.
